# A multi-label dataset for China’s agricultural and rural scenes classification from VHR satellite imagery

**DOI:** 10.1038/s41597-026-06800-8

**Published:** 2026-02-07

**Authors:** Shiying Yuan, Quanlong Feng, Bowen Niu, Xiaolu Yan, Landi Zheng, Zinuo Hao, Dehai Zhu, Jianyu Yang, Jiantao Liu

**Affiliations:** 1 College of Land Science and Technology, China Agricultural University, Beijing, 100193 China; 2 College of Resource Environment and Tourism, Capital Normal University, Beijing, 100048 China; 3 Laboratory of Geo-Information Science and Remote Sensing, Wageningen University & Research, 6708 PB Wageningen, The Netherlands; 4 Institute of Geographic Sciences and Natural Resources Research, Chinese Academy of Sciences, Beijing, 100101 China; 5 School of Surveying and Geo-Informatics, Shandong Jianzhu University, Jinan, 250101 Shandong China

**Keywords:** Environmental sciences, Agriculture

## Abstract

This study releases China-MAS-50k, i.e., China Multi-label dataset for Agriculture & rural Scene 50k, the first very-high-resolution (VHR) remote sensing dataset for multi-label classification covering entire China’s agricultural and rural areas, filling the gap in finely annotated data for non-urban scene recognition. Based on a 50 km grid system, over 50,000 sample points were determined nationwide, where VHR Google Earth imagery were to be collected for subsequent multi-label annotation. A fine-grained label system comprising 18 categories (e.g., cropland, rural village, greenhouse and photovoltaic station, etc.) was established. Meanwhile, both a rigorously defined visual interpretation system and a labeling procedure including cross-check and error correction were proposed to maintain annotation quality. Finally, the proposed dataset has a total of 55,520 VHR images with 135,289 labels, which exhibits a long-tail distribution thus providing a challenging benchmark dataset. Furthermore, we evaluated the performance of mainstream multi-label classification models on the China-MAS-50k dataset, where ResNeXt-101 achieved the best performance with an F1-score of 78.4%, but exhibited limitations in recognizing tail categories.

## Background & Summary

Agricultural and rural scenes (such as croplands, villages, ponds, greenhouses, etc.) play a critical role in the global ecological environment and have long attracted attention across various fields^1–6^. Especially for China, with its rapid economic development and continuous population growth, significant changes have taken place in the country’s agricultural and rural landscapes since the beginning of the 21st century. Particularly in the past decade, a series of national rural revitalization policies, including New Urbanization and the Beautiful Countryside Initiative, have profoundly reshaped the spatial patterns of agriculture and rural areas in China^7–9^. Therefore, there is an urgent need for up-to-date satellite remote sensing scene datasets that accurately reflect the current conditions of China’s agriculture and rural regions^10–14^.

Remote sensing scene classification refers to the automatic identification of scene types within a given image patch^15–17^. Depending on the number of scene labels to be identified, it can be categorized into single-label and multi-label classification. It is noteworthy that most current remote sensing scene recognition datasets were designed for single-label classification, such as UCM^18^, AID^19^, RSSCN7 Dataset^20^ and MSLU-100K^21^, etc. Among these, UCM^18^ is a typical dataset proposed by Newsam *et al*. at the University of California, Merced in 2010. The image size is 256 × 256 pixels, containing 21 scene categories with 100 images per category and is used for land use scene classification. AID^19^, released by Xia *et al*. in 2016, comprising 30 categories and 10,000 aerial images acquired from Google Earth, which is widely used for evaluating computer vision models in single-label scene classification. However, these datasets provide insufficient description on agricultural and rural scenes, often oversimplifying them under a single label such as “Agriculture,” thereby failing to capture the diversity and complexity of agricultural and rural scenarios.

At the same time, it is worth noting that remote sensing scenes often comprise multiple complex categories. Simply assigning a single category to an image makes it difficult to achieve fine-grained classification and modeling of real-world scenarios. To address this issue, multi-label scene recognition datasets have been introduced and gained widespread attention recently. For example, the MultiScene dataset proposed by Hua *et al*.^22^ integrates 100,000 high-resolution aerial images from six continents and 11 countries, containing 36 scene labels. The DFC15 multi-label dataset^23^ was derived from the IEEE GRSS Data Fusion Contest 2015, which consists of 3,342 aerial images with 8 categories. It should be noted that in multi-label scene datasets, the relationship between images and labels is many-to-many. As the number of labels increases, the number of possible combinations between images and labels grows exponentially, which significantly increases the difficulty of multi-label classification.

**Fig. 1:**
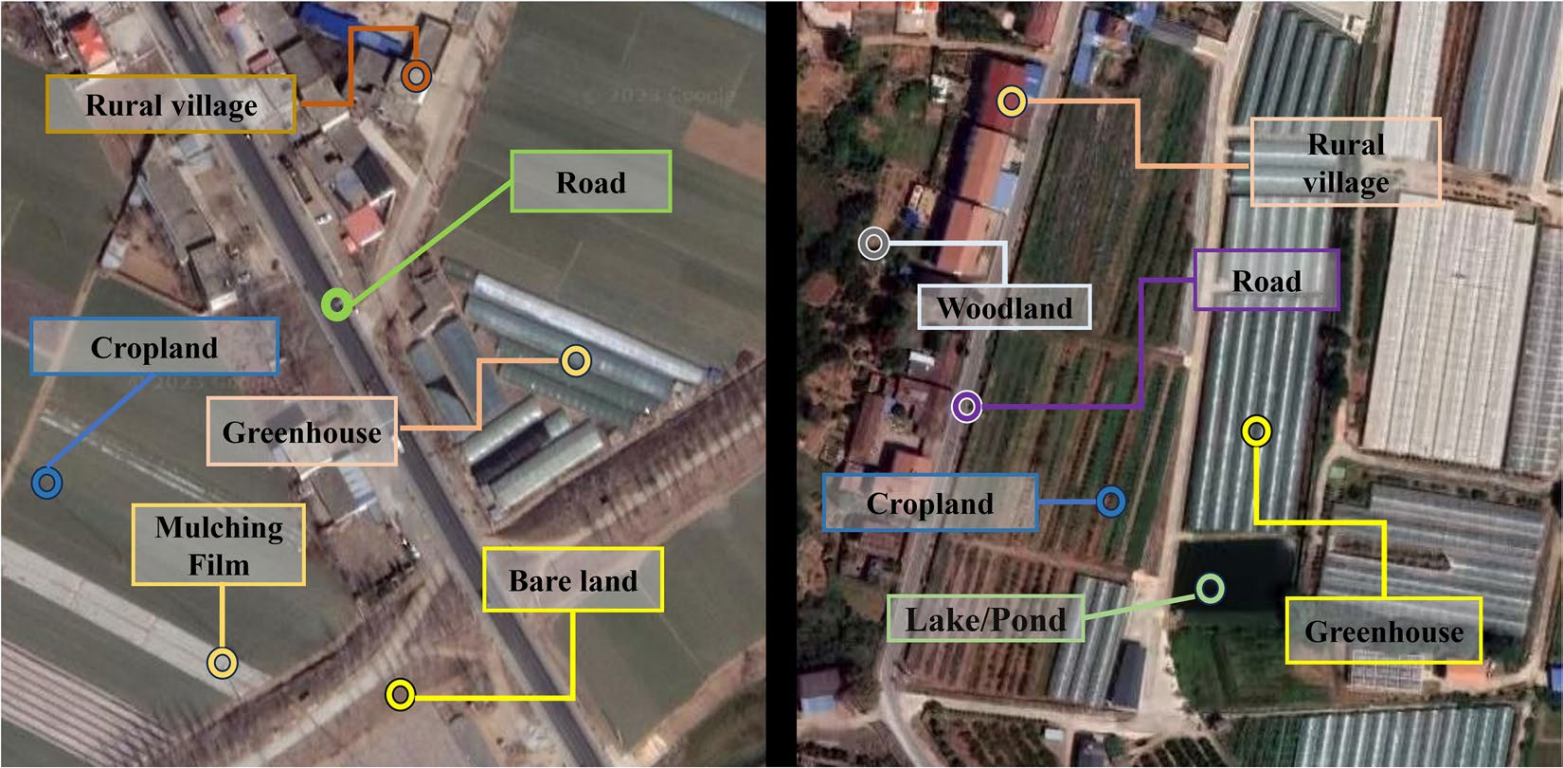
Multi-label illustration of rural scenes. The left and right image locates at 34.746°N, 118.182°E, and 30.294°N, 114.140°E, respectively. Satellite imagery copyright © Google Earth.

As mentioned above, although several multi-label scene classification datasets exist, they primarily focused on urban areas and only provided fine-grained multi-label annotations for urban scene types. However, they remain inadequate in capturing the complex and diverse landscapes of agricultural and rural regions^24–27^. To address this gap, we present the first large-scale, nationwide and finely annotated multi-label dataset for China’s agricultural and rural scenes, China-MAS-50k (China Multi-label dataset for Agriculture & rural Scene 50k), aimed at advancing the application of remote sensing multi-label classification in agricultural and rural contexts (Fig. 1). Specifically, based on VHR Google Earth imagery, we collected 55,520 sample points across China and conducted visual interpretation and multi-label annotation, generating a total of 135,289 labels. The dataset encompasses 18 categories, including cropland, woodland, grassland, rural village, greenhouse, photovoltaic stations, factory, etc. Furthermore, we systematically evaluated the performance of mainstream multi-label classification networks on the China-MAS-50k dataset, establishing a performance benchmark for multi-label recognition in agricultural and rural scenarios.

As the first nationwide, high-resolution multi-label dataset for agricultural and rural scenes, it plays a crucial role in the following research areas.

1. It serves as a critical supplement to existing multi-label classification dataset in the wild, particularly in non-urban areas, given its dedicated focus on agricultural and rural scenes.
2. It also serves as a benchmark dataset for verifying and comparing the performance of state-of-the-art multi-label classification models.
3. Weakly-supervised semantic segmentation. By leveraging multi-label clues, it enables further exploration of semantic segmentation under weak supervision, thereby achieving pixel-level classification by using only coarse scene-level labels.
4. Analysis of agricultural and rural landscape patterns in China. Since the sampling points cover the entire country and the scene labels are derived from VHR imagery, this dataset enables fine-grained characterization of China’s agricultural and rural landscapes and provides robust data support for analyzing landscape pattern differences at the national scale.

## Methods

### Overall workflow

**Fig. 2:**
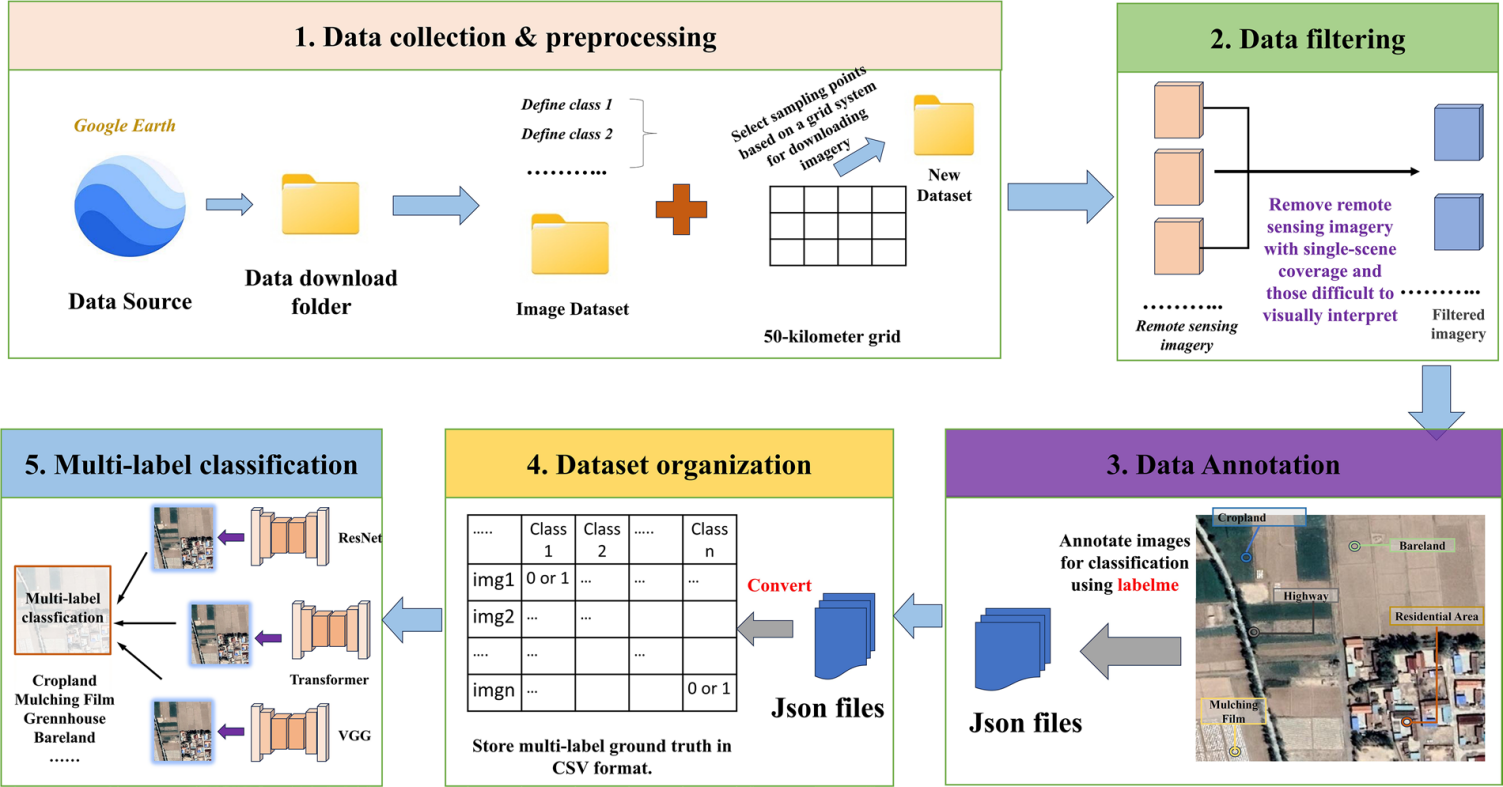
Overall workflow. Satellite imagery copyright © Google Earth.

This section describes the overall workflow of data collection, preprocessing, annotation and organization (Fig. 2). Specifically, we selected Google Earth as the source of VHR remote sensing imagery due to its open access and high image quality. Subsequently, we established a nationwide grid system with a cell size of 50 km to ensure spatial uniformity and representativeness of sampling. Based on this grid system, sample points were selected and corresponding VHR images were downloaded from Google Earth. Following this, the downloaded images underwent a filtering process to exclude those difficult to visually interpret or containing single land cover category or with a high cloud coverage. After filtering, 55,520 remote sensing images were retained. These images were then multi-label annotated through manual visual interpretation and subjected to rigorous quality checks. The annotations were organized in both JSON and CSV formats. Finally, we validated the performance of a series of state-of-the-art classification models on the proposed dataset.

### Input data

This study utilized Very-High-Resolution (VHR) satellite imagery sourced from Google Earth (http://earth.google.com/), which primarily integrates data from satellite platforms such as Airbus’s Pléiades. The Google Earth imagery we used mainly originates from Pléiades-1A and Pléiades-1B satellites and WorldView-3. To ensure spatial uniformity and representativeness, a nationwide grid system with a cell size of 50 km × 50 km was established. Sample points were visually identified within rural landscapes across these grids. VHR images were acquired at zoom level 18, corresponding to a spatial resolution of approximately 1.2 meters, with each image sized 512 × 512 pixels, and the imagery was captured during the period 2023–2024. The acquired imagery underwent a rigorous filtering process to exclude images with cloud coverage exceeding 20% of the area, as well as those affected by snow, blurring, or homogeneous land cover. After screening, a total of 55,520 high-quality images were retained for annotation. All images in this study are derived from Google Earth. For the specific code to obtain Google Earth images, please refer to https://github.com/CCCCCCYCC/multi-lable-classification.

### Data collection

**Fig. 3:**
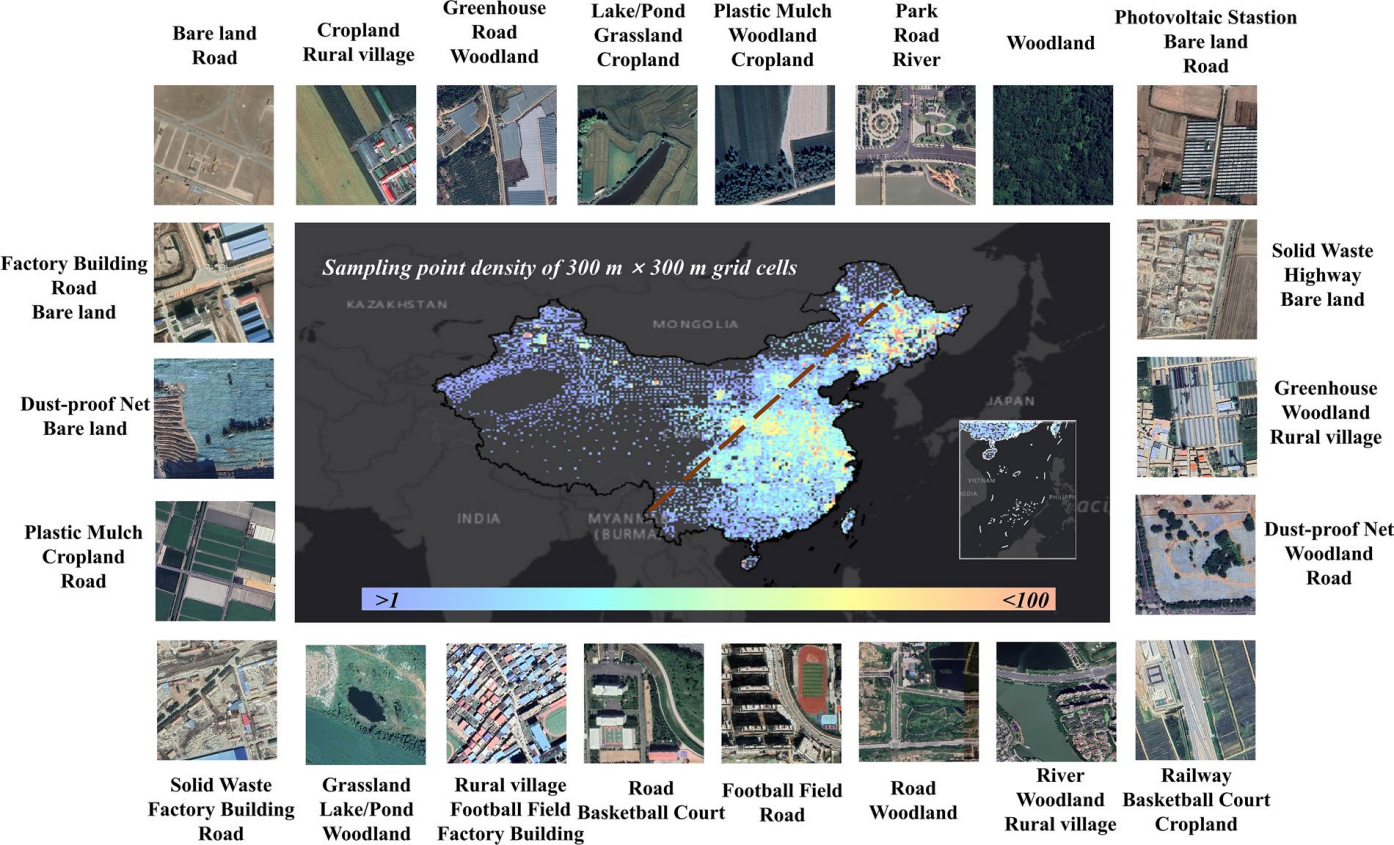
Sampling points distribution and typical examples from China-MAS-50k. Only partial labels are displayed. The diagonal line represents the Hu Line. Satellite imagery copyright © Google Earth.

The data acquisition process was performed in 2023, using the most up-to-date imagery available at that time. To ensure data quality, the downloaded images underwent a cleaning process to remove those with cloud coverage exceeding 20% of the image area, in addition to those affected by snow, blurring, and homogeneous land cover (e.g., images entirely covered by cropland), together with images difficult to interpret visually. This step was critical to guarantee high image quality and annotation accuracy. After filtering and selection, a total of 55,520 high-quality VHR satellite images covering the entire China were retained. The kernel density distribution of these sample points is shown in Fig. 3. As illustrated, most points are located to the right of the Hu Line to avoid excessive sampling of homogeneous scenes such as bare land in Northwest China. Fewer samples were collected in the Gobi Desert and the Tibetan Plateau due to their sparse and monotonous landscapes. The spatial distribution of the sample points also aligns well with the pattern of cropland distribution across China.

### Data preprocessing

Based on previous studies^28–33^ and our understanding of agricultural and rural scenes in China, we constructed a multi-label classification system with a total of 18 fine-grained scene categories: cropland, woodland, grassland, bare land, river, lake/pond, rural village, factory building, greenhouse, plastic mulch, photovoltaic station, dust-proof net, solid waste, park, football field, basketball court, road and railway.

**Fig. 4:**
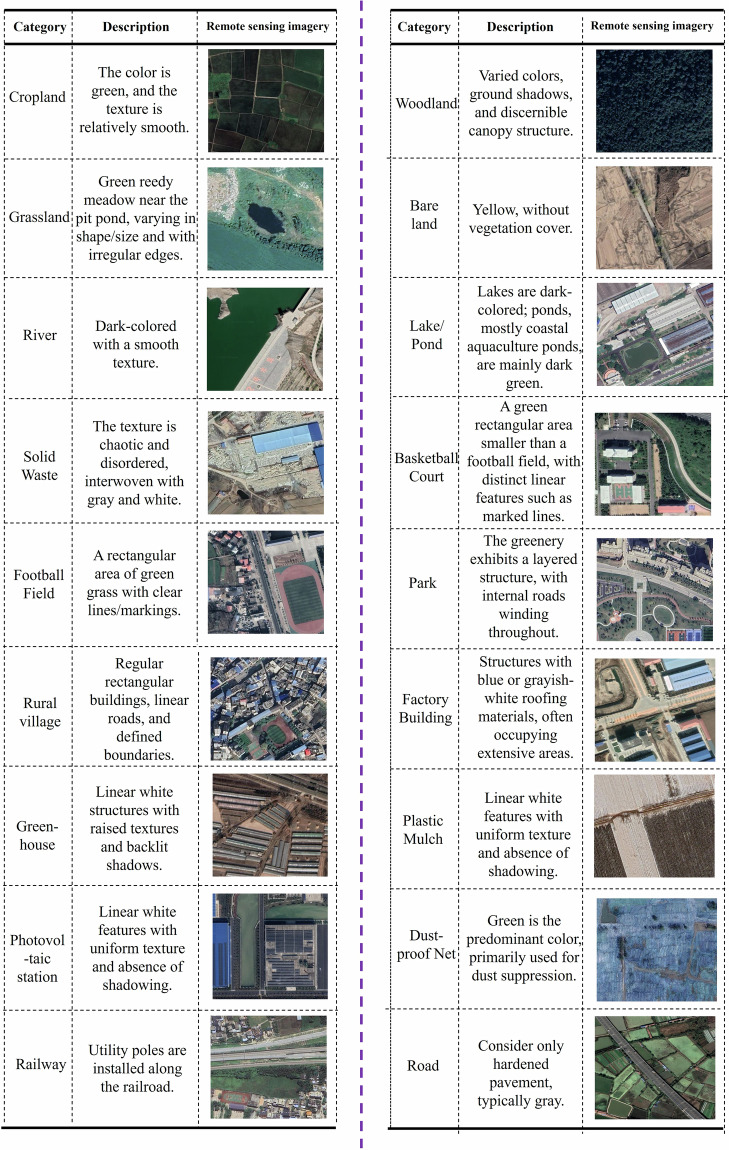
Interpretation keys based on true-color (RGB) imagery. Satellite imagery copyright © Google Earth.

Actually, in VHR remote sensing imagery, different land objects exhibit distinct characteristics such as color, shape and texture, which serve as interpretation keys for identifying complex scene types. To facilitate the multi-label annotation of agricultural and rural scenes, we established a systematic set of interpretation keys, as illustrated in Fig. 4. These keys include typical remote sensing image examples and corresponding textual descriptions to help annotators accurately understand and label each scene category. For instance, cropland typically appears green with a relatively smooth texture and clearly defined field boundaries. Bare land exhibits a yellowish tone without vegetation cover while woodland shows variations in color, displays shadow patterns indicating tree height, and can be distinguished by its textured appearance. These characteristics enable effective differentiation between cropland, bare land and woodland in VHR imagery. Similarly, greenhouses are predominantly long white structures with shadows on one side. Factory buildings usually have blue or gray-white roofs with large coverages while rural villages mostly consist of small-sized and compact individual houses. Additionally, plastic mulch appears as smooth, white elongated strips without shadows while photovoltaic stations are characterized by neatly arranged arrays of dark-gray panels with visible shadows. We also considered dust-proof nets, commonly used at construction sites in China to prevent dust pollution, which are typically made of green plastic to appear environmentally friendly. Solid waste, another category, mainly includes domestic waste piles, construction debris and industrial waste dumped near factories in rural regions.

Additionally, among the downloaded images, those depicting single-category scenes or that were uninterpretable were removed, though a small number of single-scene images were retained. To ensure sufficient samples for minority categories such as dust-proof nets, photovoltaic stations, and plastic mulch, we specifically supplemented these categories by manually identifying and recording corresponding coordinates on Google Earth. These locations were then used as additional sample points for downloading VHR satellite imagery.

### Dataset annotation and organization

**Fig. 5:**
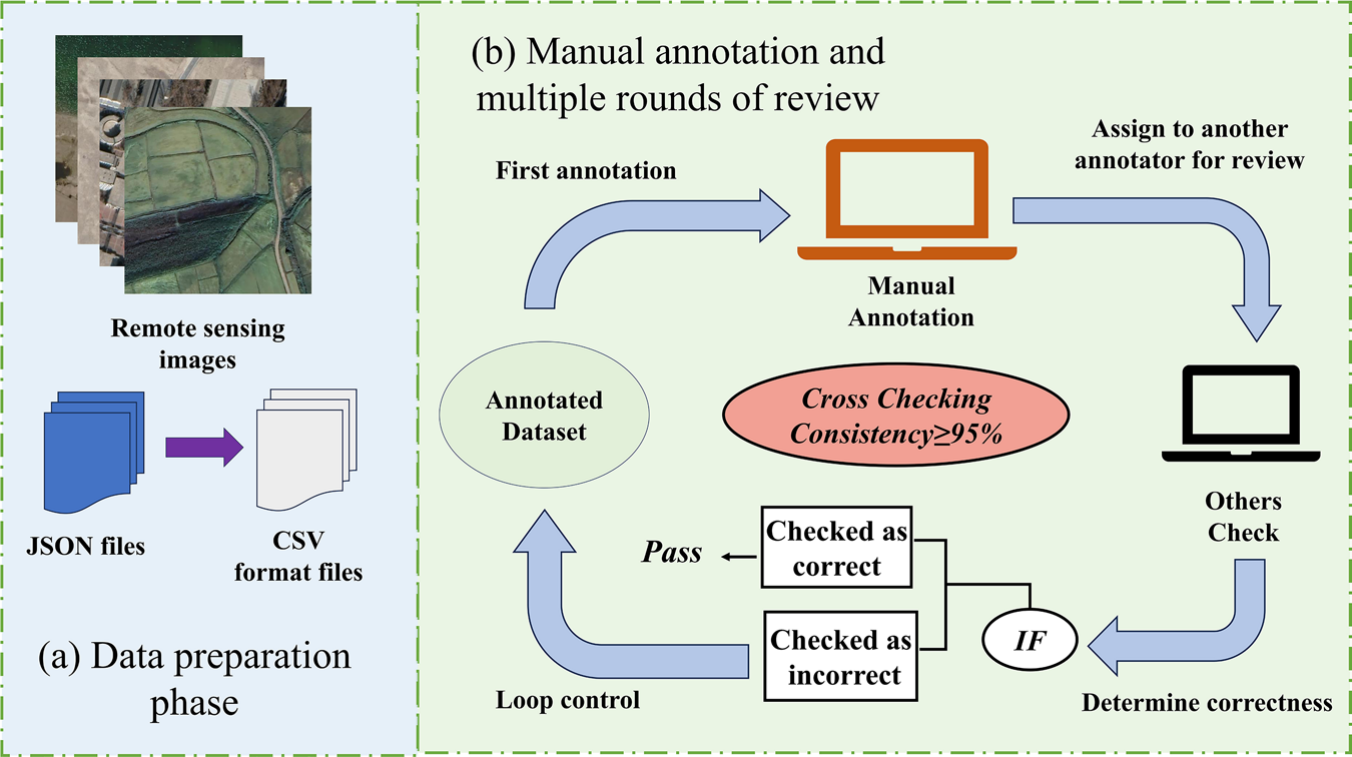
Annotation procedure. Satellite imagery copyright © Google Earth.

As mentioned above, prior to conducting the multi-label annotation task, we first established clear visual interpretation guidelines and a labeling system to ensure consistency and accuracy in the annotation process, as well as to align all annotators’ understanding of the labels. Due to its user-friendliness, versatility and open accessibility, we selected Labelme as the annotation tool for creating the multi-label dataset for agricultural and rural scenes. Using Labelme, we performed detailed multi-label annotations on the filtered set of 55,520 image patches (Fig. 5). Each scene within the image patch was annotated using the point annotation tool, and corresponding labels were created and saved. Three annotators participated in the labeling process, with emphasis on label consistency and accuracy to ensure the quality of the dataset. To further maintain annotation reliability, we conducted cross-visual inspections and made necessary corrections.

The annotated dataset is organized in a structure where satellite images are paired with multi-labels in JSON format. Furthermore, we converted the JSON files into CSV format, as the latter is more suitable for training multi-label classification models.

### Data Overview

**Fig. 6:**
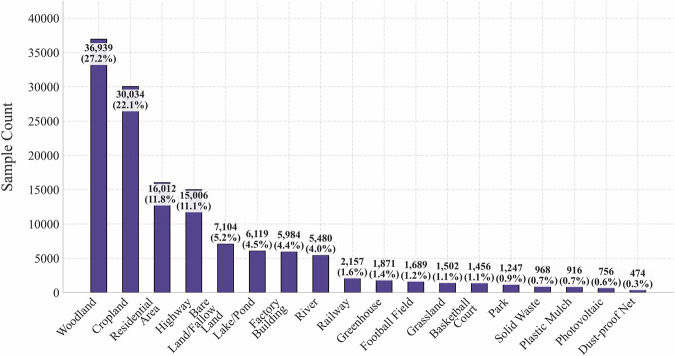
Dataset statistics.

The detailed composition of the proposed China-MAS-50k dataset is illustrated in Fig. 6. Specifically, the dataset contains a total of 55,520 VHR images together with 135,289 labels. Among them, woodland ranks first with 36,832 samples, accounting for 27.2% of the entire dataset. Cropland follows with 29,958 samples, representing 22.1% of the dataset. These two categories comprise 49.3% of all samples, which reflects the key role of forestry and farming activities in rural China. Actually, the widespread planting of trees along farmland boundaries systematically increases the number of woodland labels. In contrast, samples of modern agricultural facilities are relatively scarce, where greenhouses only account for 1,874 samples, making up 1.4% of the dataset. Besides, photovoltaic stations and dust-proof nets are the least two categories where the former has 755 samples (0.6%) while the latter comprise 471 samples (0.3%). This significant disparity in sample numbers is not a data bias but rather an authentic reflection of the reality in rural China, where the traditional natural economy remains dominant, leading to limited representation of modern agricultural infrastructure. This performance discrepancy is a core challenge posed by the long-tailed distribution and represents one of the most important application values of the dataset.

## Data Records

The proposed dataset, China-MAS-50k^34^, provides the first multi-label dataset dedicated to agricultural and rural scenes in China. The dataset consists of 55,520 VHR satellite images with 18 categories and a total of 135,289 labels, such as cropland, woodland, rural village, plastic mulch, greenhouses, photovoltaic station, dust-proof net, etc. Annotation is stored in CSV format, where a value of 1 indicates the absence of the category, while 0 indicates its presence. Besides, VHR images are stored in the image folder while in the CSV file, each column represents a category name, each row corresponds to an image filename, and a value of 0 or 1 in row i and column j indicates whether the j-th category appears in the i-th image. The dataset is openly accessible via figshare (10.6084/m9.figshare.30128272).

## Technical Validation

We evaluated the performance of existing multi-label classification models on the proposed China-MAS-50k dataset. The dataset was partitioned into training, validation and test sets with an ratio of 8:1:1. The training set was used for optimizing model parameters, the validation set for selecting the optimal model, and the test set was reserved for final accuracy evaluation after training.

### Baseline

To provide comprehensive benchmarking, we utilized typical multi-label classification models, including the classical machine learning methods such as Random Forest (RF)^34^ and XGBoost^35^, CNN-based models such as VGGNet^36^, Inceptionv3^37^, ResNet^38^, ResNeXt^39^, DenseNet^40^, as well as Transformer-based models including ViT-B/16^41^ and Swin-B^42^. All models, including traditional machine learning models and deep learning models, employ uniform input features, namely three-channel RGB images and the corresponding multi-label annotation information from the table.

### Evaluation metrics

To conduct a comprehensive evaluation, we measured the performance of typical models using label-based, example-based and overall metrics. Let $L$ and $N$ denote the number of categories and samples, respectively. The calculation methods for these metrics are as follows:

Class-based metrics. To evaluate the network performance from a category-wise perspective, we computed the mean class-based precision (*mCP*), mean class-based recall (*mCR*), mean class-based F1-score (*mCF*_*1*_), and mean per-class average precision (*mAP*). Specifically, the formulas for the metrics are as follows.

1 $$mCP=\frac{1}{L}\mathop{\sum }\limits_{c=1}^{L}\frac{T{P}_{c}}{T{P}_{c}+F{P}_{c}}$$

2 $$mCR=\frac{1}{L}\mathop{\sum }\limits_{c=1}^{L}\frac{T{P}_{c}}{T{P}_{c}+F{N}_{c}}$$

3 $$mC{F}_{1}=\frac{1}{L}\mathop{\sum }\limits_{c=1}^{L}\frac{T{P}_{c}}{T{P}_{c}+\frac{1}{2}(F{P}_{c}+F{N}_{c})}$$

4 $$mAP=\frac{1}{L}\mathop{\sum }\limits_{c=1}^{L}A{P}_{c}$$

where *TP*_*c*_, *FN*_*c*_, and *FP*_*c*_ denote the number of true positives, false negatives and false positives for category *c*, respective. The metric AP denotes the Average Precision for each individual class.

Example-based metrics. To evaluate the classification performance from a sample-wise perspective, we computed the mean example-based precision (*mEP*), mean example-based recall (*mER*), and mean example-based F1 (*mEF1*), with their formulas defined as follows.

5 $$mEP=\frac{1}{N}\mathop{\sum }\limits_{k=1}^{N}\frac{T{P}_{k}}{T{P}_{k}+F{P}_{k}}$$

6 $$mER=\frac{1}{N}\mathop{\sum }\limits_{k=1}^{N}\frac{T{P}_{k}}{T{P}_{k}+F{N}_{k}}$$

7 $$mE{F}_{1}=\frac{1}{N}\mathop{\sum }\limits_{k=1}^{N}\frac{T{P}_{k}}{T{P}_{k}+\frac{1}{2}(F{N}_{k}+F{P}_{k})}$$

where *TP*_*k*_, *FP*_*k*_, and *FN*_*k*_ denote the number of true positives, false positives and false negatives in the *k*-th sample, respectively.

Overall metrics. Overall precision (*OP*), overall recall (*OR*) and overall F1 (*OF*_*1*_) can be used to measure model performance from a more comprehensive perspective. The formulas are as follows.

8 $$OP=\frac{TP}{TP+FP}$$

9 $$OR=\frac{TP}{TP+FN}$$

10 $$O{F}_{1}=\frac{TP}{TP+\frac{1}{2}(FN+FP)}$$

where the statistics of true positives (*TP*), false positives (*FP*) and false negatives (*FN*) are based on the prediction results across all scenarios and samples.

### Validation results

To evaluate the performance of baseline models on the proposed China-MAS-50k dataset, both qualitative and quantitative validation results are given in this section.

**Fig. 7:**
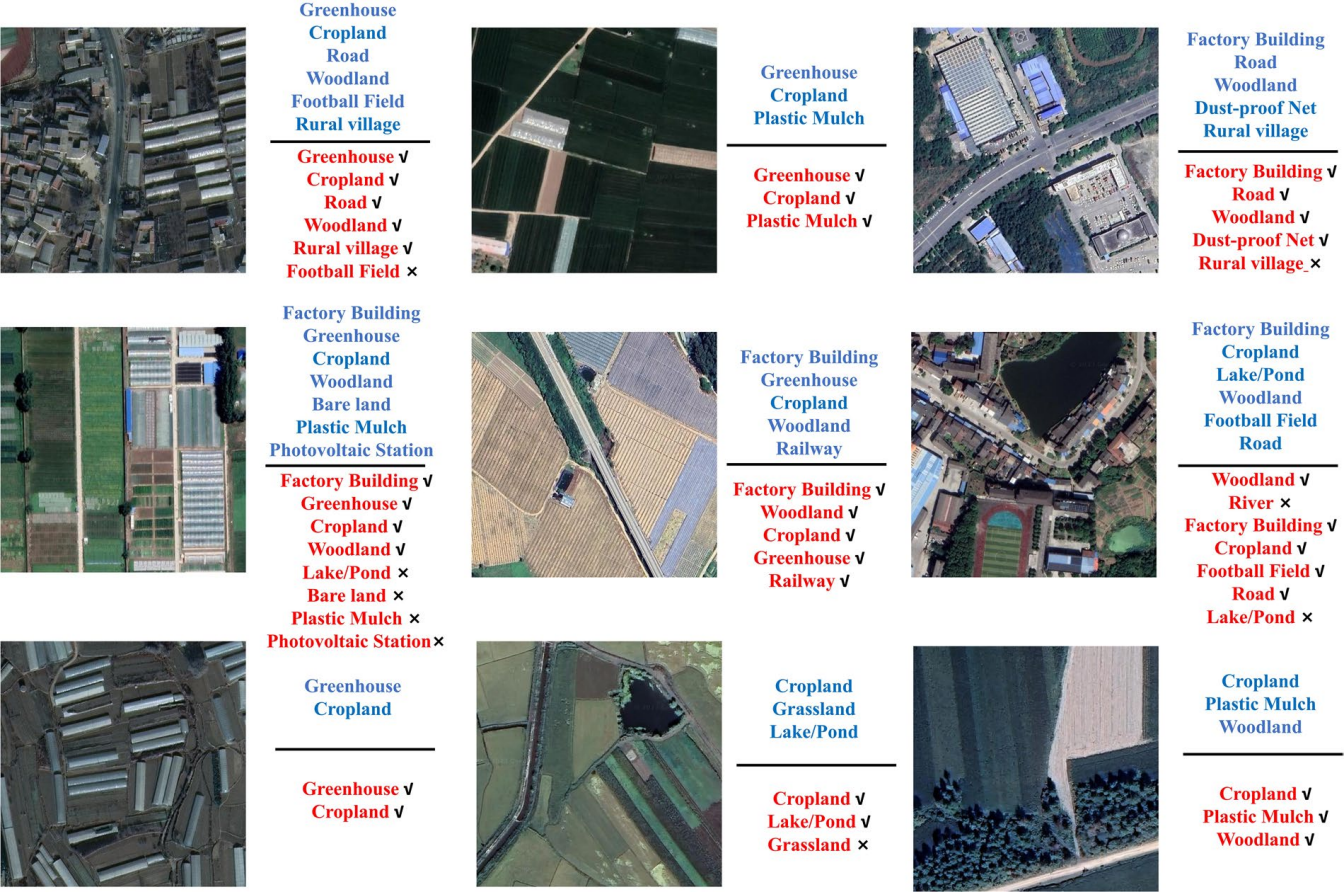
Comparison between classification results (red) and ground truth (blue), where “√” indicates correct predictions while “×” indicates misclassifications. Satellite imagery copyright © Google Earth.

Figure 7 displays several prediction results from ResNeXt-101 for reference. It can be observed that categories like cropland, woodland and road are mostly classified correctly, whereas tail categories such as photovoltaic stations and plastic mulch are misclassified. This further validates the challenging nature of our dataset, particularly for tail categories in long-tail distributions.

**Fig. 8:**
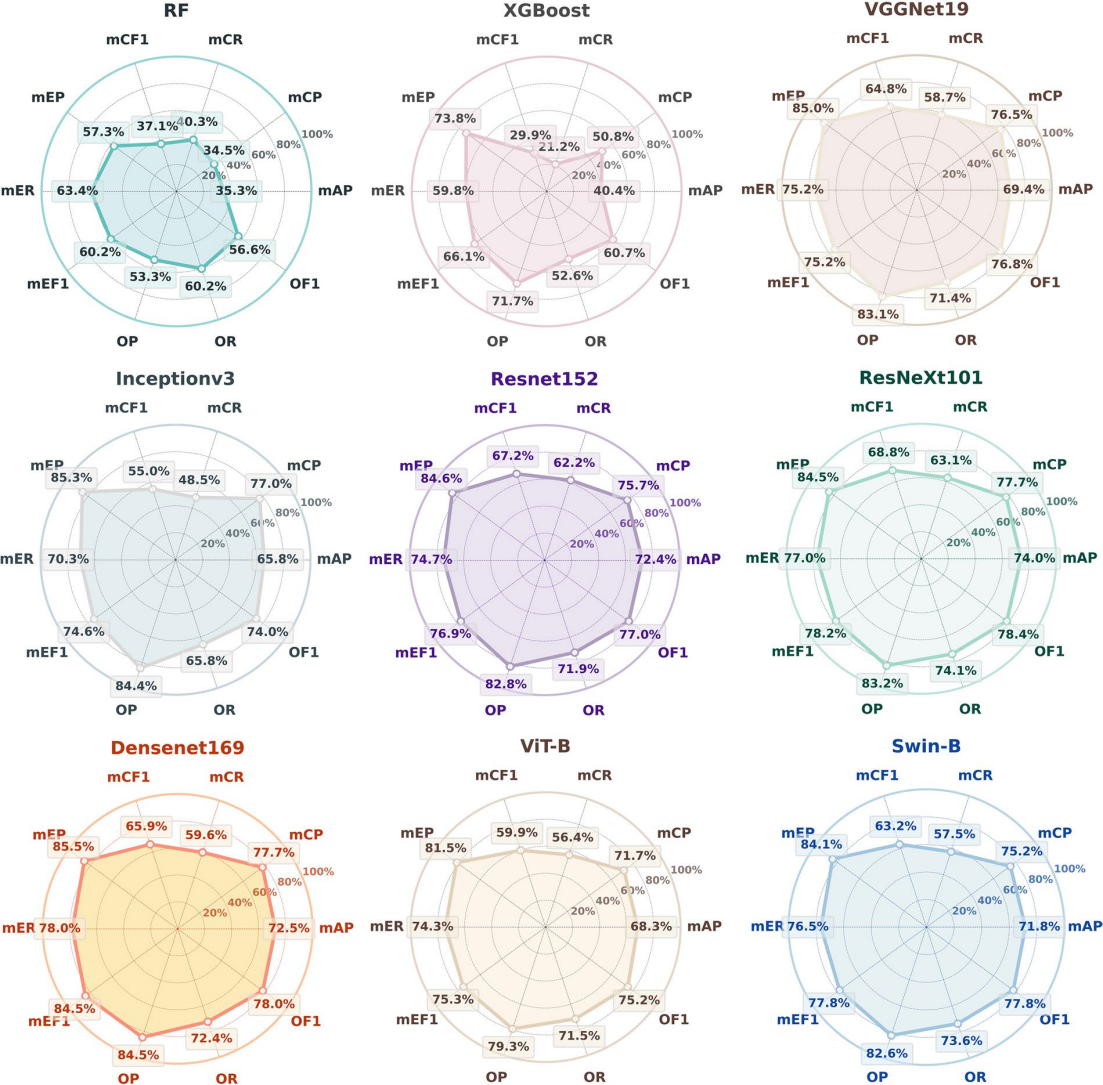
Accuracy evaluation for various baseline models.

Quantitatively, we compared several benchmark models and the accuracy metrics are shown in Fig. 8. It can be observed that ResNeXt-101 achieves the best performance in three key metrics, *mAP* (74.0%), *mCF*_*1*_ (68.8%) and *OF*_*1*_ (78.4%), demonstrating its capability and robustness from multiple perspectives. DenseNet-169 shows outstanding results in metrics such as *mAP* (78.0%) and *OF*_*1*_ (78.0%), indicating its balanced and strong performance in multi-label scene recognition. In addition, compared to traditional machine learning algorithms like RF and XGBoost, deep neural networks demonstrate significant advantages across most evaluation metrics. However, different deep learning models exhibit varying strengths and weaknesses across metrics, underscoring the impact of architectural differences on multi-label classification performance.

To further investigate the performance of each model in specific scene recognition, Table 1 presents the AP values for each category. The results show that ResNeXt-101 achieves the highest AP values in most scene categories. Meanwhile, we observe that for scenarios with scarce training samples, such as ‘greenhouses’ and ‘dust-proof nets’, the recognition accuracy of most deep learning models significantly decreases. This highlights the challenges of building unbiased models under imbalanced data distributions.

**Table 1:** Per-class AP values.

Model	CL	WL	GL	BL	RV	LP	SW	BC	FF	PK	RV	FB	GH	PM	PV	DP	RW	RD
RF	82.5	70.3	11.0	22.5	21.5	16.3	32.2	14.7	21.6	18.9	73.4	42.4	5.9	43.1	42.1	11.6	39.5	65.1
XGboost	85.5	71.3	12.5	27.6	19.6	17.1	35.1	20.8	26.7	22.4	76.9	44.3	7.3	46.8	74.3	18.7	50.4	68.0
VGG19	95.4	90.8	19.8	46.7	57.0	56.4	59.7	53.5	80.6	69.2	95.4	75.6	53.2	75.6	93.0	59.0	80.8	87.2
Inceptionv3	94.2	90.2	19.9	44.3	41.6	26.4	55.5	46.4	83.4	78.0	95.3	77.9	33.7	69.6	92.8	54,3	96.8	84.7
Densenet169	**95.8**	**92.8**	24.3	47.0	**57.0**	47.7	**65.6**	56.6	87.4	77.9	**96.8**	77.3	47.1	79.1	93.6	72.1	**98.3**	87.7
ResNet152	95.5	90.6	26.6	48.0	58.3	**55.0**	61.8	55.5	87.4	74.7	96.7	76.3	55.7	77.6	94.1	61.5	99.1	**89.4**
ResNeXt101	95.7	91.0	**30.3**	47.1	55.6	50.9	58.7	62.7	91.0	**82.3**	95.8	74.7	**60.0**	**79.6**	93.7	**76.9**	**98.3**	87.2
Swin-B	95.3	91.8	25.3	48.1	47.9	45.7	61.4	50.9	**91.4**	79.1	96.3	**81.1**	50.8	75.4	94.1	71.8	98.1	88.2
ViT-B	93.7	85.7	18.2	**51.5**	33.5	34.5	62.4	**62.8**	89.1	72.4	94.5	73.9	39.6	74.5	**95.4**	70.9	91.9	84.7

Note. CL, cropland; WL, woodland; GL, grassland; BL, bare land; RV, river; LP, lake/pond; SW, solid waste; BC, basketball court; FF, football field; PK, park; RV, rural village; FB, factory building; GH, greenhouse; PM, plastic mulch; PV, photovoltaic station; DP, dust-proof net; RW, railway; RD, road.

**Fig. 9:**
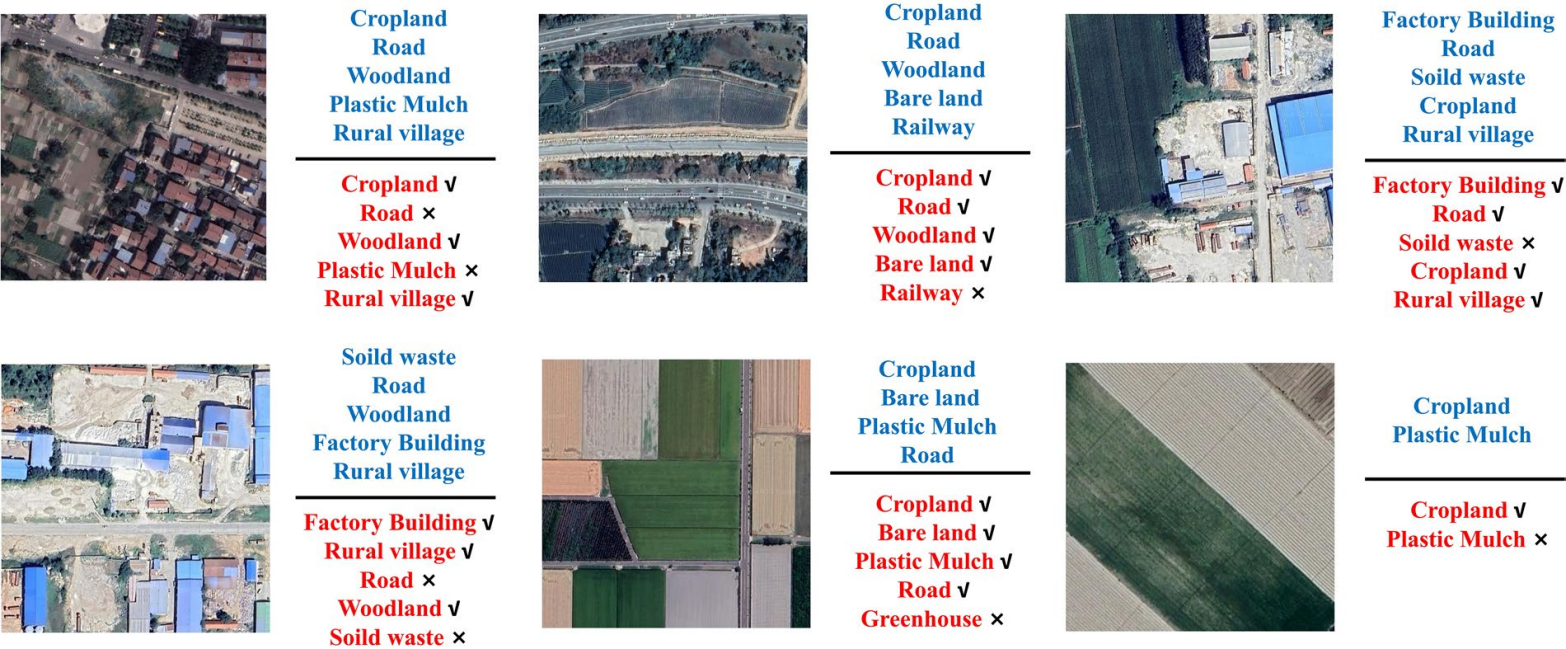
Bad cases. Satellite imagery copyright © Google Earth.

Finally, we present some cases of incorrect predictions, as shown in Fig. 9. Misclassifications primarily manifest in two forms. First, errors due to material similarity, such as confusion between plastic mulched areas and greenhouses, reflecting the model’s insufficiency in grasping of deep-level features and highlight the challenges posed by the long-tail distribution of the dataset. Second, misinterpretations of similar spatial structures. For instance, factories being mislabeled as residential areas, indicating algorithmic biases in understanding certain typical geometric patterns. More notably, the phenomenon of multi-scale target missed detection is observed. Railways, as linear and sparse targets, are absent from the correctly detected results in some cases, confirming blind spots in the model’s perception of objects with extreme aspect ratios. These errors collectively point to the current model’s limitations in representing atypical ground objects and further demonstrate the challenging nature of the proposed dataset.

## Usage Notes

The China-MAS-50k dataset is the first large-scale multi-label classification dataset focused on China’s agricultural and rural scenes, providing a foundational resource for evaluating multi-label classification models in non-urban regions. The dataset’s annotations are stored in a standard CSV format, while the remote sensing images are in PNG format.

It should be noted that the precise geographic coordinates for individual sample points are not provided due to the data collection methodology, which did not establish a one-to-one correspondence between the acquired images and their geographic locations. This limitation affects the dataset’s utility for integration with other geospatial data sources (e.g., Sentinel-2 or PlanetScope imagery) for cross-dataset analysis. Nevertheless, the core value of the dataset lies in multi-label scene recognition from image content, and this limitation does not impact its validity for model training and evaluation. The dataset exhibits a significant long-tail distribution, providing a realistic scenario for researching class imbalance problems.

Besides, the potential application of this dataset lies in extending it into an image-text multimodal dataset for rural scene understanding. Since we have annotated multiple labels within the scenes, further annotation can generate complete textual descriptions of the scenes, thereby creating a remote sensing image-text multimodal sample dataset focused on China’s agricultural and rural landscapes. For example, the following description could be generated based on the left image of Fig. 1: The scene witnesses a hardened road, with rural village situated closely on both sides. Adjacent to the rural village are extensive cropland, containing plastic mulching films and greenhouses, along with some uncultivated bare land.

Considering the substantial size of the dataset, researchers can flexibly choose to work with the complete dataset or a representative subset of it for experimentation, based on their computational resources.

## Data Availability

All data from this paper are publicly available on the figshare platform via 10.6084/m9.figshare.30128272. The compressed package contains an image folder and a CSV-format image annotation table. The dataset is straightforward and easy to use upon download. More details could be found in Data Records section.

## Code availability

The code for obtaining Google Earth images and multi-label classification on the proposed China-MAS-50k dataset is available at https://github.com/CCCCCCYCC/multi-lable-classification. The code is written in python and the deep learning framework is Pytorch. It consists all of the baseline models in this study, including Random Forest, XGBoost, VGGNet, Inceptionv3, ResNet, ResNeXt, DenseNet, ViT-B and Swin-B, together with accuracy assessment.
